# Age of Initiation of Dual Tobacco Use and Binge Drinking among Youth (12–17 Years Old): Findings from the Population Assessment of Tobacco and Health (PATH) Study

**DOI:** 10.3390/ijerph182412985

**Published:** 2021-12-09

**Authors:** Adriana Pérez, Arnold E. Kuk, Meagan A. Bluestein, Hui Min Shirlyn Sia, Baojiang Chen

**Affiliations:** 1 Department of Biostatistics and Data Science, The University of Texas Health Science Center at Houston (UTHealth), Austin, TX 78701, USA; Baojiang.Chen@uth.tmc.edu; 2 Michael & Susan Dell Center for Healthy Living, The University of Texas Health Science Center at Houston (UTHealth), Austin, TX 78701, USA; Arnold.E.Kuk@uth.tmc.edu (A.E.K.); Meagan.A.Bluestein@uth.tmc.edu (M.A.B.); Hui.Min.Shirlyn.Sia@uth.tmc.edu (H.M.S.S.)

**Keywords:** cigarette use, e-cigarette use, cigarillo use, survival analysis, interval censoring, hazard function

## Abstract

Earlier exposure to binge drinking and tobacco use is associated with higher odds of substance use disorders. Using national youth data from the PATH study, we prospectively estimate the age of initiating past 30-day use of (1) cigarettes, e-cigarettes, and binge drinking, and (2) cigarettes, cigarillos, and binge drinking. Cox proportional hazard models were used to estimate differences in the age of initiation by sex, race/ethnicity, and previous use of other tobacco products. By age 21, 4.4% (95% CI: 3.7–5.2) and 2.0% (95% CI: 1.2–2.8) of youth reported initiation of past 30-day use outcomes (1) and (2), respectively. After controlling for sex and previous use of other tobacco products, statistically significant differences in the age of initiation by race/ethnicity were found for each outcome: Hispanic and non-Hispanic Black youth were less likely than non-Hispanic White youth to initiate past 30-day use of both outcomes (1) and (2) at earlier ages. Although the initiation of both outcomes remained relatively low by age 21, these incidences represent 1.56 million and 700,000 youth, respectively. This study provides the public with evidence to identify the particular ages at which education campaigns may be most effective to prevent youth from initiating these three substances. Further research is needed to estimate the age of initiation of other dual tobacco use patterns with binge drinking.

## 1. Introduction

Tobacco and alcohol use are estimated to cause approximately 29% [1] and 5.5% [2] of cancer deaths in the U.S., respectively. Additionally, using both tobacco and alcohol further increases the risk of developing cancer. In fact, several studies show that the association between alcohol with oral and pharyngeal cancer was stronger among tobacco users, and that the joint effect of tobacco and alcohol on oral and pharyngeal cancer was not additive but multiplicative [3,4]. In addition, use of tobacco and alcohol increases the risk for adverse mental health outcomes [5,6,7,8,9]. Furthermore, the earlier people initiate tobacco and/or alcohol use, the higher the odds of developing a substance use disorder [10,11,12]. Given the greater health risk imposed by using both tobacco and alcohol, one important risk factor to consider for prevention is the age of initiation of these substances. In this manuscript, e-cigarettes are included as a tobacco product (TP) because e-cigarettes contain nicotine in the U.S. [13].

According to the 2019 National Youth Tobacco Survey (NYTS) data, e-cigarettes and cigar products were the most commonly used tobacco products among U.S. middle and high school students, with 20.0% and 5.3% middle and high school students using e-cigarettes and cigar products in the past 30-days, respectively [14]. Similarly, a previous report of the Population Assessment of Tobacco and Health (PATH) study from 2013–2016 among youth never tobacco users found that 4.7%, 6.4%, and 4.0% reported past 30-day cigarette, e-cigarette, or any cigar (cigarillos, filtered cigars, and traditional cigars) use, respectively, after one or two years of follow-up [15]. Among a nationally representative sample of youth who reported any TP use, a 2013–2014 PATH study reported the prevalence of the most common combinations of dual TP use as: 15% cigarettes and e-cigarettes and 10% cigarettes and cigarillos [16]. The same study also reported that among youth who had used any TP in the past 30-days, 43.0% reported using more than 1 TP in the past 30-days. Among these poly-tobacco users, cigarettes, e-cigarettes, and cigarillos were the most popular, with 71.4%, 53.7%, and 46.0% reporting the use of each TP [16]. Dual TP use is problematic, as it exposes youth to more tobacco toxicants than single-product use as seen in biomarker data studies [17,18], and a previous PATH study found dual TP use is associated with increased nicotine dependence [19]. Furthermore, dual use of cigarettes and e-cigarettes is associated with higher odds of cardiovascular disease than when each product is used alone [20].

A study using data from the 2017 National Survey on Drug Use and Health found that 5.3% of youth (aged 12–17) reported binge drinking in the past 30-days [21], which is a concern considering the reinforcing relationship between tobacco and alcohol, in which alcohol is primary trigger for initiation of tobacco use and relapse back to tobacco use after quit attempts [5,20,21]. Given that alcohol consumed in excessive amounts (i.e., binge drinking) and dual TP use are linked to long-term health consequences (i.e., heart diseases, stroke, cancer, and substance use disorders) [22,23,24], especially when youth start using these substances at younger ages, it is imperative to understand the age of initiation of the polysubstance use behavior of past 30-day dual TP use and binge drinking.

Sex differences in tobacco use have been reported in previous studies, with males reporting younger ages of initiation of cigarette, e-cigarette, and cigarillo use [25,26,27,28]. In addition, males are more likely to be dual and poly TP users [29]. Other studies have also found racial disparities in tobacco use patterns, with White youth reporting higher risk for all tobacco use patterns (i.e., mono, dual, and poly use) compared to their African American peers [30,31]. Similarly, sex and race/ethnicity group differences have been found in binge drinking, with male youth reporting higher rates of binge drinking than females [32], and White youth reporting higher rates of binge drinking than youth of other races/ethnicities [32].

The present paper prospectively estimates the age of initiation of two polysubstance use outcomes: past 30-day use of (i) cigarettes, e-cigarettes, and binge drinking, and past 30-day use of (ii) cigarettes, cigarillos, and binge drinking among youth (12–17 years old) in the PATH study who reported never use of these substances at their first wave of PATH participation (waves 1–4). Differences in the age of initiation of these two polysubstance use outcomes by sex and by race/ethnicity after controlling for the number of other TPs ever used were explored. Understanding the age of initiation of polysubstance use not only reinforces the importance of substance intervention at a young age, but also informs intervention and educational programs about possible differences by sex and by race/ethnicity.

## 2. Methods

### 2.1. Study Design and Participants

PATH is a nationally representative, longitudinal cohort study of US youth and adults that studies tobacco use behaviors, attitudes and beliefs, and tobacco-related health outcomes [33]. The target population of PATH consisted of individuals 12 years and older across the US, with 13,651 youth participating the study at wave 1 (September 2013 to December 2014). In addition, youth who were younger than 12 years old at wave 1 were considered “aged-up youth” when they turned 12 years old and were eligible to participate in the study in follow-up waves; 2091, 2045, 1694, and 4433 aged-up youth entered the PATH study at waves 2 (October 2014 to October 2015), 3 (October 2015 to October 2016), 4 (December 2016 to January 2018), and 5 (January 2018 to December 2019) respectively. Youth who turned 18 years old were invited to complete the adult measurements: 1915, 1907, and 1900 aged-up adults completed the adult questionnaire from waves 2 to 4 (October 2014 to January 2018), respectively. A sample of youth who reported never use of cigarettes, e-cigarettes, and cigarillos, and who did not binge drink in the past 30-days at their first wave of PATH participation (waves 1–4) were included using the restricted-use data [34]. Data from waves 2 to 5 were used to track outcomes for all participants. A total of 19,184 (representing 35,373,374 U.S. youth) constituted the sample. Informed oral consent was obtained from the parents of the youth and youth provided oral assent [33]. The Committee for the Protection of Human Subjects at the University of Texas Health Science Center at Houston provided IRB approval for this study with number HSC-SPH-17-0368.

### 2.2. Outcomes

Two polysubstance use outcomes were studied: the age of initiation of past 30-day use of (i) cigarettes, e-cigarettes, and binge drinking and the age of initiation of past 30 day use of (ii) cigarettes, cigarillos, and binge drinking. These polysubstance use outcomes are based on reporting past 30-day use of all three substances in the same PATH study wave. For each TP, PATH asked “In the past 30 days, have you smoked/used [TP], even one or two puffs/times?”. Response options included “yes”, “no”, and “don’t know”. Respondents who refused to answer or responded “don’t know” were considered missing. Among youth who reported past 30-day alcohol use, PATH also asked “On average, on those days you drank in the past 30 days, how many alcoholic drinks did you usually have each day? Count a drink as a can or bottle of beer; a wine cooler or a glass of wine, champagne, or sherry; a shot of liquor or a mixed drink or cocktail”. Boys who reported 3, 4, or 5+ drinks on days drank in the past 30 days were considered past 30-day binge drinkers for ages 12–13, 14–15, and 16–17 [35], respectively. Girls who reported 3+ drinks on days drank in the past 30 days were considered past 30-day binge drinkers [35]. Youth who answered “yes” to past 30-day cigarette use, past 30-day e-cigarette use and who reported past 30-day binge drinking in the same PATH wave were considered polysubstance users for outcome (i). Youth who answered “yes” to past 30-day cigarette use, past 30-day cigarillo use and who reported past 30-day binge drinking in the same PATH wave were considered polysubstance users for outcome (ii). Please note that cigarillo users include both blunt-only users (cigarillo users who remove the tobacco and replace it with marijuana) and those who used cigarillos as intended (see limitations section).

### 2.3. Other Tobacco Product Use

Youth may have used other tobacco products prior to initiation of each outcome. We considered other TPs, including hookah, smokeless tobacco, traditional cigars, and filtered cigars. Because of the low prevalence of use of each individual TP, we collapsed other TP use as 0 (never used other TPs) and 1+ (used at least one other TP). Youth who answered “Don’t know” to all TPs were considered missing. The number of other TPs ever used was calculated at youth’s first wave of PATH participation to ensure that the other TP use preceded each polysubstance use outcome.

### 2.4. Sex and Race/Ethnicity

The PATH study imputed self-reported participant sex by using the household information at wave 1 and categorized participants as boys and girls. The following categories measured self-reported participant race: White alone, Black alone, Asian alone, and other (including multiracial). Participants’ ethnicity was categorized as either Hispanic or non-Hispanic. In order to be comparable to the Surgeon General’s report [36], race and ethnicity were combined into four categories: non-Hispanic White, Hispanic, non-Hispanic Black, and non-Hispanic other (including, Asian and multiracial participants).

### 2.5. Age of Initiation of Polysubstance Use Outcomes

Two separate prospective estimations were conducted: (i) the age of initiation of past 30-day use of cigarettes, e-cigarettes, and binge drinking and (ii) the age of initiation of past 30-day use of cigarettes, cigarillos, and binge drinking. PATH provided the age of youth in years at each wave and the number of weeks between survey waves. We used participants’ age at their first wave of PATH participation (waves 1–4) and the number of weeks between survey waves (waves 2–5) until each past 30-day use outcome (i and ii) to estimate the lower and upper age bounds as an interval-censored age of initiation. For all participants, the lower age bound was the highest possible age a participant remained a non-polysubstance user for each outcome. The lower age bound was calculated as the sum of the age at the first wave of PATH participation and the number of weeks between survey waves until the latest wave where the participants reported non-use of all three substances in the past 30-days. For those who become users, the upper age bound was the highest possible age when youth first reported past 30-day use of each polysubstance use behavior (i and ii). The upper age bound was calculated as the sum of the age at their first wave of PATH participation and the number of weeks between survey waves until the first report of using all three substances in the past 30-days for each outcome. For youth who remained non-users of all three substances for each outcome, the upper age bound was censored.

### 2.6. Statistical Analysis

PATH uses a complex survey design, requiring the use of sampling weights and balanced repeated replicate weights with Fay’s correction factor of 0.3 [33,37]. Weighted summary statistics are provided. The distribution of the age of initiation for polysubstance use outcomes (i) and (ii) were estimated using weighted interval-censoring survival analysis [38]. The hazard function (and its 95% confidence intervals) for each polysubstance use outcome was estimated using the Turnbull non-parametric estimator [39], and are reported as cumulative incidence in percentages, which is presented in a figure. Weighted interval-censoring Cox proportional hazards regression models [40] were used to examine differences in the estimated age of initiation by sex and by race/ethnicity while controlling for ever use of other TPs. If there were statistically significant differences by sex or by race/ethnicity on the age of initiation of each polysubstance use outcome, weighted interval-censoring survival analyses were conducted stratified by sex or by race/ethnicity. There was very little missingness in PATH, and missing values are reported. All statistical analyses were conducted using SAS version 9.4 (SAS Institute, Cary, NC, USA).

## 3. Results

### 3.1. Sample Demographics

Demographic characteristics of PATH youth who had never used cigarettes, e-cigarettes, and cigarillos and had not binge drank in the past 30-days are reported in Table 1. Their average age was 13.6 years old, 55.2% entered the study at wave 1, 51% were males, and 23.4% were Hispanic. Among youth, 0.2%, 0.02%, 1.3%, and 0.7% have ever used traditional cigars, filtered cigars, hookah, or smokeless tobacco, respectively. Therefore, 2.2% had ever used any other TP.

### 3.2. Prospective Estimation of Age of Initiation of Past 30-Day Polysubstance Use Outcomes

Table 2 shows the distribution of the age of initiating past 30-day polysubstance use outcomes for the entire sample of youth. Initiation of both polysubstance use outcomes remained low for the ages that characterize youth (12–17 years). However, by age 21, 4.44% (95% CI: 3.72–5.16) of youth are estimated to initiate past 30-day use of cigarettes, e-cigarettes, and binge drinking, representing 1.57 million U.S. youth. By age 21, 1.98% (95% CI: 1.20–2.77) of youth are estimated to initiate past 30-day use of cigarettes, cigarillos, and binge drinking, representing over 700,000 U.S. youth.

### 3.3. Crude and Adjusted Hazard Ratios of the Age of Initiation of Past 30-Day Polysubstance Use Outcomes

Table 3 presents the crude hazard ratios estimating differences in the age of initiation of each polysubstance use outcomes by sex, by race/ethnicity, and by ever use of other TPs. Table 3 also presents the adjusted hazard ratios (AHRs) estimating differences in the age of initiation of each polysubstance use outcome, while controlling for sex, race/ethnicity, and ever use of other TPs simultaneously. Hispanic youth were 43% less likely (AHR: 0.57; 95% CI: 0.41–0.80) and Non-Hispanic Black youth were 85% less likely (AHR: 0.15; 95% CI: 0.06–0.35) to initiate past 30-day use of cigarettes, e-cigarettes, and binge drinking at earlier ages compared to Non-Hispanic White youth. Hispanic youth were 48% (AHR: 0.52; 95% CI: 0.32–0.86) less likely, Non-Hispanic Black youth were 64% (AHR: 0.36; 95% CI: 0.14–0.91) less likely, and Non-Hispanic Other were 65% (AHR: 0.35; 95% CI: 0.13–0.96) less likely to initiate past 30-day use of cigarettes, cigarillos, and binge drinking at earlier ages compared to Non-Hispanic White youth. However, no statistical differences in initiating each past 30-day polysubstance use outcome were observed for sex in both crude and adjusted analyses.

**Figure 1:**
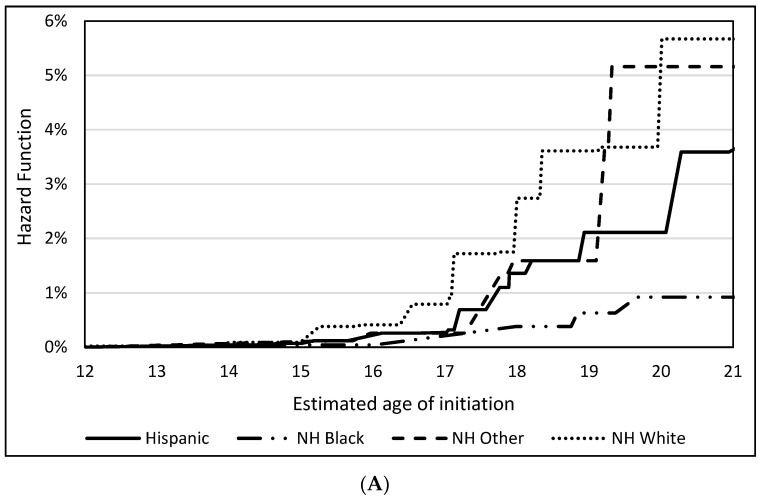

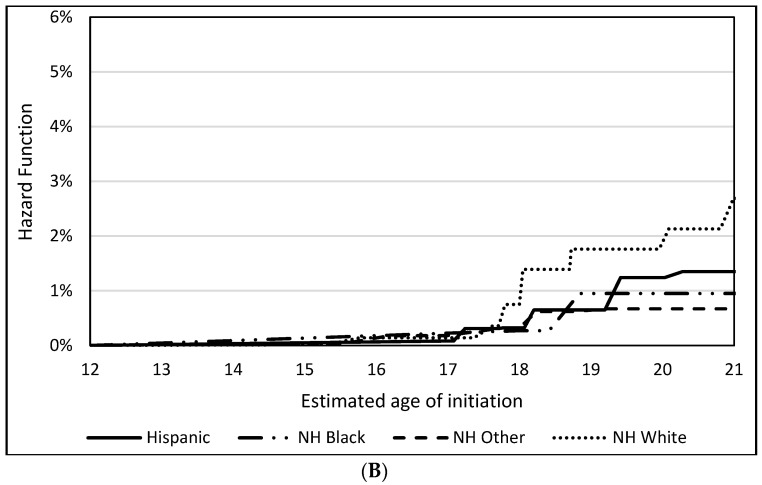
Hazard functions of the age of initiation of past 30-day use of (**A**) cigarettes, e-cigarettes, and binge drinking and past 30-day use of (**B**) cigarettes, cigarillos, and binge drinking by race/ethnicity.

Figure 1 shows the hazard functions of the age of initiating each polysubstance use outcome stratified by race/ethnicity. Overall, Non-Hispanic White youth were estimated to initiate both past 30-day polysubstance use outcomes at earlier ages, only surpassed by Non-Hispanic Other youth between ages 19 and 20 for cigarettes, e-cigarettes, and binge drinking. However, by age 21, Non-Hispanic White youth were estimated to have the highest probability of initiation of past 30-day polysubstance use of cigarettes, e-cigarettes, and binge drinking (5.7%), followed by Non-Hispanic Other youth (5.2%), Hispanic youth (3.7%), and Non-Hispanic Black youth (0.9%). By age 21, 2.7% of Non-Hispanic White youth were estimated to initiate past 30-day polysubstance use of cigarettes, cigarillos, and binge drinking, followed by Hispanic youth (1.4%), Non-Hispanic Black youth (0.9%), and Non-Hispanic other youth (0.7%).

## 4. Discussion

This study is the first to prospectively estimate the age of initiation of past 30-day polysubstance use of cigarettes, e-cigarettes, and binge drinking, as well as the age of initiation of past 30-day polysubstance use of cigarettes, cigarillos, and binge drinking. Reporting past 30-day polysubstance use of three substances in the same wave of PATH is a risky behavior in youth and becomes even more problematic when this occurs at younger ages, despite the fact that it was illegal for youth to purchase TPs before 18 and alcohol before 21 years old. The Tobacco 21 Law went into effect in December 2019 [41], which did not affect our participants. While initiation of each polysubstance use outcome remained relatively low by age 20, the estimated percentages of initiation of past 30-day polysubstance use of cigarettes, e-cigarettes, and binge drinking represent over 1.56 million youth and past 30-day polysubstance use of cigarettes, cigarillos, and binge drinking represent over 700,000 youth, which is problematic. Adolescence represents a period of critical growth and development during which neurobiological, physical, emotional, and social changes occur in youth [42], and the use of these three substances has implications for these developments.

Our findings are similar to previous studies on the relationship between TP and alcohol use. The 2014 Monitoring the Future study reported the odds of initiating past 30-day alcohol use among those who were past 30-day poly-nicotine TP users as 19.7 (AOR: 13.6–28.5) for 8th and 10th graders and as 11.5 (AOR: 8.4–15.7) for 12th graders [43]. Our study prospectively followed-up never users while the previous study reported cross-sectional analyses which included users and non-users. Our study goes beyond this previous work by estimating the age when youth initiate past 30-day use of all three substances in the same PATH wave.

A study based on the 2003 National Youth Risk Behavior Survey that defined binge drinking as consuming more than five drinks in a row during the past 30-days found that among 12–20 year old respondents, 17.8% of 12–14 year olds, 28.9% of 15–17 year olds, and 38.7% of 18–20 year olds were binge drinkers [44]. Our results are similar to this study, finding that binge drinking is more common in 18+ year olds compared to 12–14 and 15–17 year olds [44]. This is concerning as a prior study suggests that binge drinking in late adolescence predicts binge drinking at ages 30–31 [45].

Our study found that Hispanic and Non-Hispanic Black youth had decreased risk of earlier ages of initiating past 30-day polysubstance use outcomes compared to Non-Hispanic White youth. This is consistent with the current literature finding that White high school students often report earlier initiation of alcohol use [32,46]. Similarly, data from nationally representative surveys found that Non-Hispanic White youth were more likely to initiate e-cigarette use, and had higher prevalence of exclusive, dual and poly tobacco use than youth of other racial/ethnic groups [47,48], which is in line with our findings. However, studies have not successfully documented possible reasons as to why this racial/ethnic difference occurs in e-cigarette use [49,50]. Future studies should investigate such factors to determine the reasons for this disparity. Our results found that Non-Hispanic Black youth are less likely to initiate past 30-day use of cigarettes, cigarillos, and binge drinking at earlier ages compared to Non-Hispanic White youth. In contrast, a recent study that found that Non-Hispanic White youth were less likely to initiate past 30-day use of cigarillos as a single TP at earlier ages than Non-Hispanic Black youth [25]. This inconsistency could arise from the confounding effects due to concurrent use of cigarettes and binge drinking, substances which studies have shown Non-Hispanic White youth initiate earlier [29,34,48].

Previous publications of PATH youth reported the age of initiation single TPs, including cigarettes [27], e-cigarettes [26], and cigar products [25], which found that males are more likely to initiate past 30-day use of cigarettes, e-cigarettes, or cigar products at earlier ages than females. This is contrary to our results where we did not observe differences in the age of initiating polysubstance use between males and females. The reasons for this are unclear and should be explored in future research, as other studies also have shown that males were more likely than females to engage in binge drinking [32] and that males are at higher risk of dual TP use compared to females [29].

There are many complications associated with dual tobacco use and binge drinking in youth, which are compounded the earlier that youth initiate use of these substances, such as drinking and driving [51], more frequent use of heroin and methamphetamines [51], risky sexual behavior [52], and alcohol poisoning [52], among others. A recent e-poster presentation at a 2021 Tobacco Regulatory Science meeting hosted by the National Institutes of Health argued for the necessity of communicating cancer risks associated with alcohol and tobacco use and showed that messages with images warning of the health risks of using both tobacco and alcohol are more effective than messages without images. This suggests that prevention and education programs that use messages containing images warning the health risks associated with using both tobacco and alcohol may be necessary [53].

Strengths of this study include the longitudinal prospective analysis of the age of initiation of past 30-day polysubstance use of three substances using a nationally representative sample across PATH waves 1–5. One of the limitations of our study is that we depended on self-reported data for the cigarette, e-cigarette, cigarillo, and binge drinking status to estimate the age of initiation. In addition, we use interval censoring survival analysis because asking participants the exact date they initiated all three substances is unrealistic. Another limitation is that due to the survey design of the cigar variables in PATH, it is not possible to separate blunt-only users. The variable that asks about blunt use asks about using traditional cigars, cigarillos, or filtered cigars as blunts in a single question. Then, past 30-day cigarillo use is asked without specifying if they are blunt users. However, considering that cigarillo users are still exposed to nicotine and tobacco toxicants even after replacing the tobacco in cigarillos with marijuana [54], the implications from this study should still stand.

## 5. Conclusions

In conclusion, this study prospectively estimates the age of initiating past 30-day use of (1) cigarettes, e-cigarettes, and binge drinking, and (2) cigarettes, cigarillos, and binge drinking, and identifies Non-Hispanic White youth as being at a higher risk of initiating the aforementioned polysubstance use at earlier ages in comparison to Hispanic youth or Non-Hispanic Black youth. This study provides interventionists and the public with evidence to identify the particular ages at which education campaigns may be most effective to prevent youth from initiating past 30-day polysubstance use and to target the specific racial/ethnic group that is the most vulnerable.

## Figures and Tables

**Table 1:** Demographic characteristics of PATH ^¥^ youth (aged 12–17) never users at their first wave of participation.

Total		n = 19,184; N = 35,373,374 ^1^	
		n (N)	Weighted % (SE)
Wave of Entry into PATH	Wave 1 (2013–2014)	10,725 (19,526,422)	55.2 (54.5–55.9)
	Wave 2 (2014–2015)	1958 (3,861,663)	10.9 (10.5–11.4)
	Wave 3 (2015–2016)	1891 (3,900,349)	11.0 (10.6–11.5)
	Wave 4 (2016–2017)	4610 (8,084,939)	22.9 (22.3–23.5)
Age at entry into study (SE)	Weighted mean (SE)	13.60 (13.58–13.61)	
Sex	Female	9369 (17,466,088)	49.38 (48.7–50.1)
	Male	9809 (17,893,133)	50.58 (49.9–51.3)
	Missing	6 (14,153)	0.04 (0.02–0.09)
Race/ethnicity	Non-Hispanic White	9032 (18,562,779)	52.5 (51.8–53.2)
	Hispanic	5646 (8,292,879)	23.4 (22.9–24.0)
	Non-Hispanic Black	2653 (4,875,757)	13.8 (13.3–14.3)
	Non-Hispanic Other ^2^	1811 (3,567,876)	10.1 (9.7–10.5)
	Missing	42 (74,083)	0.2 (0.1–0.3)
Ever use of traditional cigars	Yes	31 (64,086)	0.2 (0.1–0.3)
	No	19,109 (35,228,543)	99.6 (99.5–99.7)
	Missing	44 (80,744)	0.2 (0.2–0.3)
Ever use of filtered cigars	Yes	5 (8018)	0.02 (0.01–0.06)
	No	19,179 (35,365,356)	99.98 (99.94–99.99)
	Missing	0	
Ever use of hookah	Yes	255 (461,349)	1.3 (1.1–1.5)
	No	18,902 (34,861,413)	98.6 (98.3–98.8)
	Missing	27 (50,612)	0.1 (0.09–0.21)
Ever use of smokeless tobacco	Yes	140 (253,798)	0.7 (0.6–0.9)
	No	18,905 (34,859,059)	98.5 (98.3–98.7)
	Missing	139 (260,517)	0.7 (0.6–0.9)
Ever use of other tobacco products	0	18,766 (34,607,864)	97.8 (97.6–98.1)
	1+	418 (765,510)	2.2 (1.9–2.4)

^¥^ Population Assessment of Tobacco and Health (PATH) Study data reprinted with permission from the United States Department of Health and Human Services (National Addiction & HIV Data Archive Program, 2021). Restricted file received disclosure to publish: 21 July 2021; ^1^ n = sample size, N = estimated population size; ^2^ Non-Hispanic Other includes Asian, multi-race, and etc.

**Table 2:** Estimated hazard functions ^1^ (with 95% confidence intervals) of the age of initiation of each past 30-day polysubstance use outcome in PATH ^¥^ youth (2013–2019).

Age of Initiation	Past 30-Day Polysubstance Use Outcomes (95% CI)	
	E-Cigarettes, Cigarettes, and Binge Drinking	Cigarillos, Cigarettes, and Binge Drinking
12	0.0%	0.0%
13	0.0%	0.0%
14	0.02 (0.00–0.07)	NA ^2^
15	0.07 (0.02–0.11)	0.02 (0.00–0.05)
16	0.37 (0.12–0.63)	0.10 (0.04–0.17)
17	0.50 (0.27–0.73)	0.10 (0.00–0.21)
18	1.39 (0.92–1.87)	0.50 (0.10–0.90)
19	2.45 (2.01–2.88)	1.23 (0.91–1.55)
20	3.56 (2.63–4.49)	1.52 (1.07–1.98)
21	4.44 (3.72–5.16)	1.98 (1.20–2.77)

^¥^ Population Assessment of Tobacco and Health (PATH) Study data reprinted with permission from the United States Department of Health and Human Services (National Addiction & HIV Data Archive Program, 2021). Restricted file received disclosure to publish: 21 July 2021; ^1^ Hazards are reported as cumulative percentages (i.e., cumulative incidence); ^2^ NA: Not available. There was not enough sample size to produce a stable estimate of the HR.

**Table 3:** Crude and adjusted hazard ratios (with 95% confidence intervals) for the age of initiation of past 30-day polysubstance use outcomes in PATH ^¥^ youth (2013–2019).

	Past 30-Day Use of Cigarettes, E-Cigarettes, and Binge Drinking	Past 30-Day Use of Cigarettes, Cigarillos, and Binge Drinking
Crude hazard ratios (95% CI)		
Sex		
Female	1.00	1.00
Male	0.85 (0.66–1.09)	1.21 (0.78–1.88)
Race/ethnicity		
Non-Hispanic White	1.00	1.00
Hispanic	**0.58 (0.42–0.80)**	**0.52 (0.32–0.86)**
Non-Hispanic Black	**0.15 (0.06–0.35)**	**0.36 (0.14–0.91)**
Non-Hispanic Other ^1^	0.78 (0.44–1.37)	**0.35 (0.13–0.95)**
Ever use of other tobacco products		
0	1.00	1.00
1+	1.34 (0.73–2.47)	1.22 (0.46–3.25)
**Adjusted hazard ratios (95% CI)**		
Sex		
Female	1.00	1.00
Male	0.84 (0.65–1.09)	1.18 (0.76–1.83)
Race/ethnicity		
Non-Hispanic White	1.00	1.00
Hispanic	**0.57 (0.41–0.80)**	**0.52 (0.32–0.86)**
Non-Hispanic Black	**0.15 (0.06–0.35)**	**0.36 (0.14–0.91)**
Non-Hispanic Other ^1^	0.78 (0.44–1.36)	**0.35 (0.13–0.96)**
Ever use of other tobacco products		
0	1.00	1.00
1+	1.36 (0.73–2.51)	1.22 (0.46–3.21)

^¥^ Population Assessment of Tobacco and Health (PATH) Study data reprinted with permission from the United States Department of Health and Human Services (National Addiction & HIV Data Archive Program, 2021). Restricted file, received disclosure to publish: 21 July 2021 and 28 July 2021; ^1^ Non-Hispanic Other includes Asian, multi-race, and etc.; Bolded numbers represent statistical significance using a type I error level of 5%

## Data Availability

All the data from waves 1–5 are available from the Population Assessment of Tobacco and Health (PATH) Study [United States] Restricted-Use Files. Inter-university Consortium for Political and Social Research [distributor], https://doi.org/10.3886/ICPSR36231.v28, accessed on 20 November 2021. Researchers can apply for access to the restricted-use datasets from the Inter-university Consortium for Political and Social Research (ICPSR) at the University of Michigan. To access data in the Virtual Data Enclave (VDE), a Restricted Data Use Agreement (RDUA) must be established between the University of Michigan and the researcher’s institution. Data are provided via ICPSR’s VDE. For further information, please reference the VDE Guide to learn about the application process, about using the VDE, and how to request disclosure review of VDE output located here: https://www.icpsr.umich.edu/web/pages/NAHDAP/vde/index.html, accessed on 20 November 2021. Obtaining results using the restricted-use datasets requires a disclosure process with protocols set by ICPSR. When a researcher logs on to the VDE, a virtual machine is launched on the researcher’s own desktop but operates from a server at ICPSR. The virtual machine is isolated from the researcher’s physical desktop computer—users cannot download or upload files or parts of files from or to the VDE; print VDE contents to a printer; or email, copy, or otherwise move files in or out of the VDE computing environment, either accidentally or intentionally. Results are only disclosed by ICPSR after programs have been checked for accuracy and results have been replicated.
